# Feasibility of pharmacokinetic parametric PET images in scaled subprofile modelling using principal component analysis

**DOI:** 10.1016/j.nicl.2021.102625

**Published:** 2021-03-13

**Authors:** Débora E. Peretti, Remco J. Renken, Fransje E. Reesink, Bauke M. de Jong, Peter P. De Deyn, Rudi A.J.O. Dierckx, Janine Doorduin, Ronald Boellaard, David Vállez García

**Affiliations:** a University of Groningen, University Medical Center Groningen, Department of Nuclear Medicine and Molecular Imaging, The Netherlands; b University of Groningen, University Medical Center Groningen, Cognitive Neuroscience Centre, Department of Biomedical Sciences of Cell & Systems, The Netherlands; c University of Groningen, University Medical Center Groningen, Department of Neurology, Alzheimer Research Centre, The Netherlands; d University of Antwerp, Institute Born-Bunge, Laboratory of Neurochemistry and Behaviour, Belgium

**Keywords:** Alzheimer’s disease, Disease pattern, Pharmacokinetic modelling, Pittsburgh compound B, SSM/PCA

## Abstract

Scaled subprofile model using principal component analysis (SSM/PCA) is a multivariate analysis technique used, mainly in [^18^F]-2-fluoro-2-deoxy-d-glucose (FDG) PET studies, for the generation of disease-specific metabolic patterns (DP) that may aid with the classification of subjects with neurological disorders, like Alzheimer’s disease (AD). The aim of this study was to explore the feasibility of using quantitative parametric images for this type of analysis, with dynamic [^11^C]-labelled Pittsburgh Compound B (PIB) PET data as an example. Therefore, 15 AD patients and 15 healthy control subjects were included in an SSM/PCA analysis to generate four AD-DPs using relative cerebral blood flow (*R*_1_), binding potential (*BP*_ND_) and SUVR images derived from dynamic PIB and static FDG-PET studies. Furthermore, 49 new subjects with a variety of neurodegenerative cognitive disorders were tested against these DPs. The AD-DP was characterized by a reduction in the frontal, parietal, and temporal lobes voxel values for *R*_1_ and SUVR-FDG DPs; and by a general increase of values in cortical areas for *BP*_ND_ and SUVR-PIB DPs. In conclusion, the results suggest that the combination of parametric images derived from a single dynamic scan might be a good alternative for subject classification instead of using 2 independent PET studies.

## 1 Introduction

Proper interpretation of positron emission tomography (PET) scans is important for clinical diagnosis, and to monitor disease progression and response to treatment (Lammertsma, 2017). This interpretation is often made through visual inspection of the images or by means of semi-quantitative approaches such as standardized uptake values (SUV) or a ratio (SUVR), when there is a reference region without specific binding of the tracer. However, these measurements have proven to be deceiving in some cases. For example, visual assessment relies on the reader’s expertise and is prone to inter-reader disagreement (Borczyskowski et al., 2006, Morbelli et al., 2015). Meanwhile, semi-quantitative methods might result in an incorrect estimation of tracer binding, since they fail to capture the complex exchange of influx, retention, and clearance of the radiotracer between plasma and tissue of interest (Lammertsma, 2017). Previous studies, such as longitudinal measurements of amyloid load in Alzheimer’s disease (AD) patients (van Berckel et al., 2013) and neurokinin-1 receptor status after the administration of an agonist (Wolfensberger et al., 2011), have illustrated the difference between measuring tracer uptake semi-quantitatively and measuring a pathophysiologic process quantitatively (Lammertsma, 2017). For this, and to obtain an optimal quantification of the (patho)physiology under study, it is necessary to decompose the PET signal into its different components, or kinetic ‘states’ (Carson, 2003), for example, in a compartment that expresses tracer concentration that is specifically bound to the target and a separate compartment with free tracer in tissue (Gunn et al., 2001). These quantitative metrics can be obtained by applying pharmacokinetic modelling to PET data. Furthermore, pharmacokinetic models can be applied to the whole PET dataset at a voxel-level, resulting in high-quality parametric images that can be used to perform a visual assessment, with the potential to reduce misclassification and improve the inter-reader agreement, and to accurately quantify tracer uptake (Collij et al., 2019, Lammertsma, 2017, Peretti et al., 2019c).

In the case of AD, current research guidelines require the assessment of abnormal deposits of amyloid-β (Aβ), scattered through brain grey matter (Thal et al., 2002), for the classification of a patient in the so-called ‘Alzheimer spectrum’ (Jack et al., 2018). This can be done *in vivo*, for example, by the use of ^11^C-labelled Pittsburgh Compound B (PIB) PET scans. The quantification of these Aβ deposits can be obtained by means of pharmacokinetic modelling of the tracer using the simplified reference tissue model 2 (SRTM2) (Peretti et al., 2019a, Yaqub et al., 2008), which provides a measure of Aβ load through binding potential (*BP*_ND_), as well as information on regional cerebral blood flow (rCBF) through the relative tracer flow parameter (*R*_1_) (Chen et al., 2015, Meyer et al., 2011, Peretti et al., 2019c, Peretti et al., 2019b). Previous studies have shown that rCBF is closely related to glucose metabolism measured with [^18^F]-2-fluoro-2-deoxy-d-glucose (FDG) PET (Jueptner and Weiller, 1995), another PET radiotracer used routinely for the classification of AD patients and, therefore, has been suggested as an alternative to performing two scans (Meyer et al., 2011, Peretti et al., 2019c, Peretti et al., 2019b). This approach is of great interest since it might reduce patient discomfort, exposure to radiation, and study costs. Therefore, pharmacokinetic modelling of a single dynamic PIB PET scans might provide information closely related to data generated by both static FDG and static amyloid PET scans, with the further advantage of using quantitative data.

Accurate detection at the initial stages or at risk of developing neurological diseases is of major importance to develop new therapeutic strategies that aim at preventing disease progression (Garcia-Ptacek et al., 2016, Teune et al., 2010). To better understand the underlying pathophysiology in AD and its progression over time, several research groups rely on the use of mass univariate statistical techniques for image data analysis, such as statistical parametric mapping (SPM). While these methods are useful to identify differences between groups, they might not have sufficient power to explore some of the subtle brain alterations that frequently occur in neurological disorders (Alexander and Moeller, 1994). Therefore, other multivariate approaches for analysis of functional brain images are gaining interest, such as graph theory (Bullmore and Sporns, 2009, Sánchez-Catasús et al., 2017), dynamic causal modelling (Friston et al., 2003), scaled subprofile model (Meles et al., 2015, Peng et al., 2014, Spetsieris et al., 2009), and independent component analysis (ICA) (Pagani et al., 2017).

More specifically, the voxel-based scaled subprofile model analysis based on principal component analysis (SSM/PCA) is a technique that is able to generate disease-related patterns Furthermore, it quantifies the disease expression of a new subject’s image compared to this pattern by giving a score (Alexander and Moeller, 1994, Moeller and Strother, 1991), which can be used to assess how much the patient expresses the disease pattern or not. This technique has been applied mostly to FDG PET scans (Meles et al., 2017, Meles et al., 2015, Spetsieris et al., 2009, Teune et al., 2014a, Teune et al., 2013) and especially in Parkinson’s disease (Kogan et al., 2019, Peng et al., 2014, Spetsieris et al., 2013). Nonetheless, images provided by different radiotracers have been used as input for SSM/PCA type of analysis (Campbell et al., 2013, Lilja et al., 2018), and studies involving other neurological disorders, such as AD, have been explored (Eidelberg, 2009, Teune et al., 2014a, Teune et al., 2010). However, to the knowledge of the authors, only images that show the semi-quantitative total uptake of the tracer have been used in this type of analysis. Parametric images derived from pharmacokinetic analysis of dynamic PET scans might provide more accurate depictions of the disease (Collij et al., 2019). Moreover, the use of combined parametric datasets from a single dynamic PET study for the classification of AD patients using SSM/PCA has not yet been described. Finally, the use of different radiotracers and images may provide additional information about the pathophysiology of the disease through different biomarkers and may support studies focused on disease staging.

In this study, the feasibility of using parametric images derived from pharmacokinetic modelling as input for SSM/PCA analysis was explored together with the necessary changes for this application to be implemented, with a single dynamic PIB PET scan as an example. Results provided using these quantitative parametric images were compared to those obtained from the semi-quantitative SUVR images of independent PIB and FDG PET studies to investigate their level of agreement. This study provides the first step for future research studies using parametric images derived from pharmacokinetic modelling in SSM/PCA in clinical settings.

## 2 Materials and methods

### 2.1 Subjects

A cohort of 79 subjects was drawn from a larger ongoing study at the Memory Clinic of the University Medical Center Groningen (UMCG), Groningen, The Netherlands. Subjects were selected based on their clinical diagnosis revised after PET imaging, which will be further explained in this section. All subjects gave their written informed consent to participate in the study, which was approved by the Medical Ethical Committee of the UMCG (2014/320). Standard dementia screening was performed for all subjects, including the mini-mental state examination (MMSE) (Pangman et al., 2000). A minimum MMSE score of 18 was considered for subjects to be mentally competent to give their consent to participate in the study. The minimum MMSE score for this cohort was 19. Healthy control (HC) volunteers presented MMSE scores above 28 and no cognitive complaints. The study was conducted in agreement with the Declaration of Helsinki and subsequent revisions.

Multimodal neuroimaging was performed, including PIB and FDG PET, and T1-3D magnetic resonance imaging (MRI), for all included patients and control subjects. Patients were diagnosed by consensus in a multidisciplinary team based on clinical assessment according to the guidelines of the National Institute on Aging Alzheimer’s criteria (NIA-AA) (McKhann et al., 2011) for the AD patients, on the Petersen criteria (Petersen et al., 2001) for the mild cognitive impairment (MCI) subjects, on the Third Report of the dementia with Lewy bodies (DLB) consortium (McKeith et al., 2017) for the DLB patients, and on all the variants of frontal temporal dementia (FTD) (Gorno-Tempini et al., 2011, Harris et al., 2013, Rascovsky et al., 2011) for the FTD patients. Next, patients’ diagnoses were reconsidered by a review board of experienced neurologists and nuclear medicine physicians also taking into account visual assessment of the PET and MRI images acquired: AD diagnosis was based on the National Institute on Aging and the Alzheimer’s Association Research Framework (Jack et al., 2018); MCI subjects were divided into MCI+ or MCI−, according to whether the subjects presented Aβ deposition in grey matter brain tissue or not, based on visual inspection of the PIB PET scans. In total, 1 DLB and 6 FTD patients had their diagnoses changed to AD and were, therefore, included in the AD group. Table 1 presents a summary of the demographic characteristic of all subjects.

**Table 1:** Demographic characteristics of subjects included in this study. The reported p-values resulted from an ANOVA comparing the groups. (AD = Alzheimer’s Disease, MCI+ = Mild Cognitive Impairment with Aβ deposition, MCI− = Mild Cognitive Impairment without Aβ deposition, HC = Healthy Control, FTD = Frontal Temporal Dementia, DLB = Dementia with Lewy Bodies, MMSE = Mini-Mental State Examination, n = number of subjects).

		AD (n = 24)	MCI+ (n = 14)	MCI- (n = 12)	HC (n = 18)	FTD (n = 5)	DLB (n = 6)	*p*-value
Sex	Male	16	7	10	13	3	3	
	Female	8	7	2	5	2	3	
Age (years)		67 ± 7	66 ± 5	65 ± 9	68 ± 4	69 ± 6	70 ± 8	0.5
MMSE Score		24 ± 3	27 ± 2	27 ± 2	30 ± 1	28 ± 2	23 ± 4	<0.01

### 2.2 PET acquisition

All subjects underwent a dynamic PIB PET and a static FDG PET scans. PET was acquired using either a Siemens Biograph 40mCT or 64mCT scanner (Siemens Medical Solution, USA) that were harmonized regarding their performance and reconstructions. There were no statistically significant differences between data acquired from different scanners (Peretti et al., 2019c, Peretti et al., 2019b). Radiotracers were synthesized at the department of Nuclear Medicine and Molecular Imaging of the UMCG, according to Good Manufacturing Practice, and were administered via a venous cannula. Both scans were performed under standard resting conditions with eyes closed. All PET images were reconstructed from list-mode data using 3D OSEM (3 iterations and 24 subsets), point spread function correction, and time-of-flight. The resulting images had a matrix of 400 × 400 × 111, with isotropic 2-mm voxels, and smoothed 2-mm Gaussian filter at full width and half maximum (FWHM).

Dynamic PIB PET acquisition started 10 s before injection (379 ± 51 MBq) and lasted at least 60 min (frames: 7 × 10 s, 3 × 30 s, 2 × 60 s, 2 × 120 s, 2 × 180 s, 5 × 300 s, and 2 × 600 s). The static FDG PET images started 30 min after tracer injection (203 ± 7 MBq), lasted for 20 min, and were preferably performed on the same day, with the FDG PET scan occurring at least 90 min after PIB injection. However, 23 subjects had a delay, ranging from 1 to 5 months between scans. This delay showed no effect on the resulting scores.

### 2.3 Image processing

Image registration and data analysis were performed using PMOD (version 3.8; PMOD Technologies LLC). Firstly, the T1-3D images were spatially normalized to the Montreal Neurologic Institute (MNI) space using three tissue probability maps (Ashburner and Friston, 2005). Secondly, the dynamic PIB PET images were corrected for motion, using the average of its first 12 frames as reference. Thirdly, the motion corrected PIB and the FDG PET scans were aligned to the individual’s MRI, and then transformed to the MNI space using the parameters obtained with the T1-3D image. The Hammers atlas (Hammers et al., 2003) was used to draw a volume of interest (VOI) containing only the grey matter of the cerebellum. Finally, all images were smoothed using a 6-mm Gaussian filter at FWHM and all voxels outside the brain were masked out of the image.

*R*_1_ and *BP*_ND_ parametric images were generated using pharmacokinetic modelling of the dynamic PIB PET images using SRTM2 (Wu and Carson, 2002) in a voxel-based approach (Yaqub et al., 2008). The grey matter of the cerebellum VOI was used as the reference region due to the absence of specific binding of the radiotracer in this tissue (Joachim et al., 1989, Klunk et al., 2004, Price et al., 2005, Yamaguchi et al., 1989). This frequently used model is based on a two-step approach: firstly an estimation of *R*_1_, *BP*_ND_, and *k*_2_′ is done using the simplified reference tissue model (SRTM) (Lammertsma and Hume, 1996); secondly, the *k*_2_′ parameter is fixed as the median value of all voxels that presented a *BP*_ND_ estimation of 0.5 or above (Peretti et al., 2019a); and then, the model is fitted again using the results from the first run as input, generating the final *R*_1_ and *BP*_ND_ parametric maps.

To compare with *R*_1_ and *BP*_ND_ from dynamic PIB PET studies, SUVR images were generated for FDG as well as for PIB PET scans. To this end, the dynamic PIB PET scans were converted into static images by averaging the frames that corresponded to uptake times of 40–60 min. Voxel values were divided by the average value of the grey matter of the cerebellum.

### 2.4 Scale subprofile modelling/principal component analysis

SSM/PCA was applied to each set of parametric images (quantitative set of *R*_1_ and *BP*_ND_, and a semi-quantitative of FDG-SUVR, and PIB-SUVR) using in-house software implementing the SSM/PCA procedure based on a previously published study (Spetsieris et al., 2009) and validated against this original work (Teune et al., 2010). A whole brain mask was applied to remove voxels outside the brain. Therefore, this analysis was restricted to only voxels within the brain. In addition, a value of 0.001 was set as a threshold for minimum voxel value in all images. This step ensured that regions where the pharmacokinetic modelling might have failed due to the lack of specifically bound PIB PET signal, or voxels that did not show any tracer uptake, would not affect the analysis. Only *BP*_ND_ images were affected by this threshold. The excluded voxels were found mainly in the ventricles and remaining voxels outside the brain such as cerebral spinal fluid and veins, and a range between 16% and 48% of voxels were excluded depending on the subject. Intensity normalization was not performed because all parametric images are quantitative and, by definition, already normalized to the reference region. Then, an offset was removed from the data by subtracting the mean value across HC subjects, per voxel. Next, a principal component analysis (PCA) was performed, and the principal components (PC) were ranked accordingly to their explained variance of the data. The set of PCs that combined explained at least 50% of all data variance were selected (Spetsieris et al., 2009). A stepwise forward combination method was used to create a pattern; the pattern with the lowest Akaike information criteria (AIC) was chosen as the final disease-related pattern (DP). Each subject received a score by taking the inner product of the DP and the subject’s image, which reflected how much their PET scan resembled the DP. Furthermore, a leave-one-out cross validation (LOOCV) was performed to verify the stability of the DPs in this study. For each subject left out, a new DP was generated using the remaining subjects and the left-out subject received a new score based on this DP.

For the generation of the DP, 15 HC subjects and 15 AD patients were randomly selected. The same set of subjects was used for the construction of the DP for each image type. The remaining subjects (9 AD, 14 MCI+, 12 MCI-, 5 FTD and 6 DLB patients, and 3 HC subjects) were tested against the generated DP and received a score of their resemblance to the AD-DP. All final scores were standardized to a Z-score using the mean and standard deviation of the HC group based on the combined LOOCV and the test scores.

The DP generated by the *R*_1_ maps were compared to the one from FDG-SUVR and *BP*_ND_ DP, to PIB-SUVR DP using joint histograms with a base 10 logarithmic amplitude scale. Correlation between the DPs was further explored using a linear regression model. In these comparisons, the quantitative parametric maps were considered the independent variable and the semi-quantitative SUVR images, the dependent. This configuration allowed for an exploration of how much the metabolism explains the regional blood flow, and how much of the PIB uptake is reflected in the binding of the tracer.

### 2.5 Statistical analysis of the scores

Receiver-operating characteristic (ROC) curves were created to find a score threshold that would best differentiate HC from AD subjects with the highest sensitivity and specificity based on Youden’s method (Youden, 1950). Visual assessment of the images by experienced clinicians was used as the reference for diagnosis. This analysis was performed using the LOOCV scores of the AD and HC subjects used for generating the pattern. With the remaining 9 AD patients and 3 HC subjects’ scores, sensitivity and specificity of these thresholds were tested.

An ANOVA per method was performed to explore whether the scores of the groups of patients with different diagnoses were significantly distinct. The *p*-values were adjusted for multiple comparisons using the Tukey method. The difference between group means and the 95% confidence interval (CI) will be reported along with the *p*-value. All analyses were performed using RStudio (version 1.1.463, R version 3.5.2) (R Development Core Team, 2018). A corrected *p*-value of 0.05 was used as significance threshold for all analyses.

## 3 Results

### 3.1 Disease patterns

**Fig. 1:**
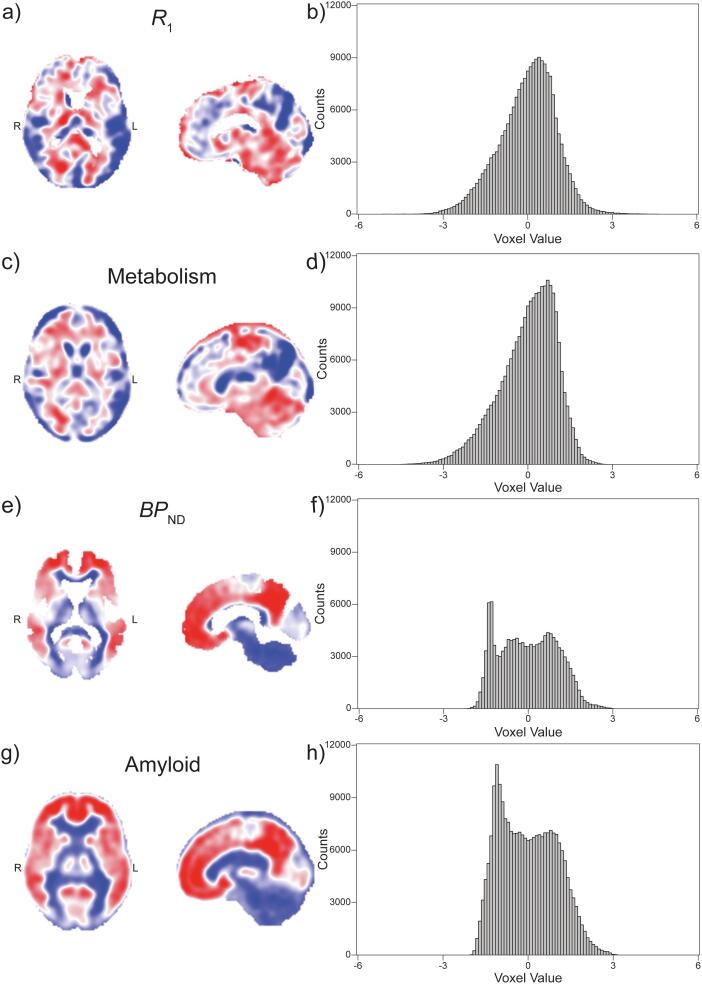
Disease Patterns and Histograms. On the first column, DPs resulted from the comparison between HC subjects and AD patients for (a) PIB-*R*_1_, (c) FDG-SUVR, (e) PIB-*BP*_ND_, and (g) PIB-SUVR. Negative voxel values correspond to blue pixels, while positive voxels values are depicted in red. The whiter the voxel colour, the closer to zero its value. All colour scales were adjusted to the same range. The second column shows the corresponding histograms of the DPs (b) PIB-*R*_1_, (d) FDG-SUVR, (f) PIB-*BP*_ND_, and (h) PIB-SUVR. The ranges of the histograms were adjusted to the same values so that it is easier to compare *R*_1_ and FDG-SUVR DP counts, and *BP*_ND_ with PIB-SUVR. (For interpretation of the references to colour in this figure legend, the reader is referred to the web version of this article.)

The DPs contained voxel values that showed the differences between AD patients and HC subjects (Fig. 1). DP voxels with negative values indicated regions where there was a decrease in the parameter (e.g. *R*_1_ or SUVR) for AD patients compared to the HC group and vice versa for the positive values.

#### 3.1.1 *R*_1_ disease pattern

When deriving the DP for the *R*_1_ images, it was found that the first six PCs together accounted for 53.26% of the explained variance of the data. The final disease pattern was generated using a linear combination of components 1 and 3, which provided the best distinction between the AD patients and the HC subjects and together explained 26.91% of the variance. The AD pattern generated was characterized by decreased perfusion in the parietal, temporal, and frontal lobes, while there was an increase in perfusion in the white matter and grey matter of the cerebellum (Fig. 1a). The histogram of the image (Fig. 1b) showed a unimodal distribution of voxel values, slightly skewed to the left.

#### 3.1.2 Metabolism FDG-SUVR disease pattern

When deriving the DP for FDG-SUVR images, it was found that the first five components together accounted for 55.62% of the explained variance of the data. The final disease pattern was generated using the first component alone, which provided the best distinction between AD patients and HC subjects and explained 20.98% of the variance. The metabolic AD pattern visually resembled the *R*_1_ DP, with a large decrease of metabolism in the parietal and frontal lobes (Fig. 1c). The histogram of this image (Fig. 1d) also showed a unimodal distribution moderately skewed to the left.

#### 3.1.3 Amyloid *BP*_ND_ disease pattern

The first component accounted for 58.12% of the explained variance of the PIB *BP*_ND_ data. Therefore, the final DP was composed of this component alone. This pattern was characterized by a general increase of amyloid deposition in brain grey matter of AD patients in comparison to HC subjects (Fig. 1e). Furthermore, a reduction in deposition can be seen in the occipital lobe. The histogram of this image’s voxel values (Fig. 1f) shows a multimodal distribution that is skewed to the right.

#### 3.1.4 Amyloid PIB-SUVR disease pattern

The first component alone accounted for 61.01% of the explained variance of the data. Consequently, the final DP was composed of this single component. This pattern was marked by an increase of Aβ deposition in grey matter all over the brain of AD patients when compared to HC subjects, while a decrease can be observed in white matter (Fig. 1g). The histogram of the voxel values from this image (Fig. 1h) presented a bimodal distribution skewed to the right.

### 3.2 Joint histograms of the disease patterns

**Fig. 2:**
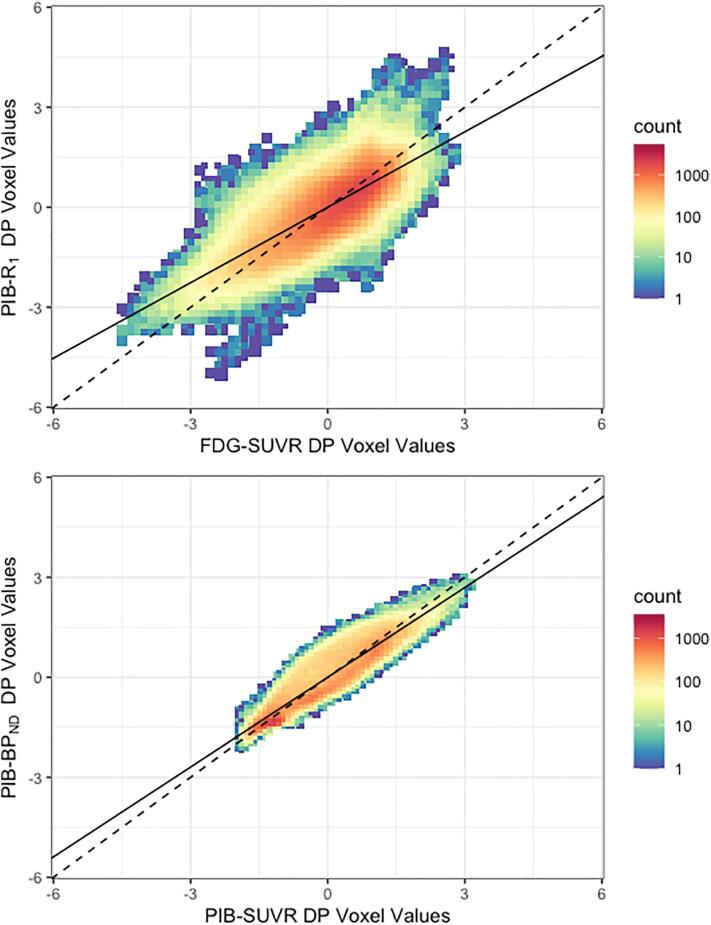
Joint histograms of the Disease Patterns. Joint histograms of the FDG-SUVR and PIB-R_1_ (top) and (bottom) PIB-SUVR and PIB-*BP*_ND_ DPs. The dashed line corresponds to the identity line, and the solid line, to the linear regression of the data from the DPs. The bin counts are displayed in base 10 logarithmic scale.

Fig. 2 top shows the joint histogram of the FDG-SUVR DP versus the *R*_1_ DP, and shows a high correlation between the two patterns, of 0.76. In addition, the FDG-SUVR DP presented a good predictability of the *R*_1_ DP, accounting for 58% of the variability (slope = 0.75, intercept = 0, *R*^2^ = 0.58, *p* < 0.001). Meanwhile, Fig. 2 bottom shows the joint histogram of the PIB-SUVR DP versus the *BP*_ND_ DP, which shows an even higher correlation between the patterns, of 0.92. The PIB-SUVR DP accounted for 86% of the variance of the *BP*_ND_ DP (slope = 0.9, intercept = 0, *R*^2^ = 0.86, *p* > 0.001).

### 3.3 Threshold score

Using the ROC curves, it was possible to derive an SSM/PCA score threshold for classifying AD versus HC. For the *R*_1_ parametric images, this threshold was of 0.01 (specificity = 1, sensitivity = 0.9) and had an area under the curve (AUC) of 0.81. FDG-SUVR threshold score was of 1.3 (specificity = 0.75, sensitivity = 1) and had an AUC of 0.91. Furthermore, *BP*_ND_ resulted in a threshold score of 3.59 (specificity = 0.6, sensitivity = 1) with an AUC of 1. Finally, the threshold score for PIB-SUVR was 3.225 (specificity = 0.6, sensitivity = 1), with an AUC of 1.

### 3.4 Distribution of scores

**Fig. 3:**
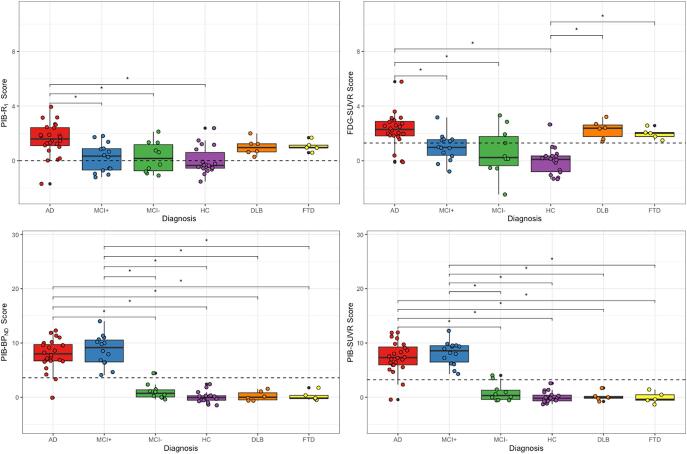
Distribution of Scores per Diagnosis. Distribution of the subjects’ Z-scores from PIB-R_1_ (top left), FDG-SUVR (top right), PIB-*BP*_ND_ (bottom left), and PIB-SUVR (bottom right). Boxes represent the interquartile range of score distribution; the horizontal line, the median score per group; the whiskers expand up to 1.5 times the interquartile range; and the remaining black dots correspond to outliers. Coloured circles represent the subject scores within the groups. Dashed lines correspond to the threshold for classifying subjects as AD. The stars represent the differences between the groups that are statistically significant. AD = Alzheimer’s Disease, MCI+ = Mild Cognitive Impairment with Amyloid deposition, MCI− = Mild Cognitive Impairment without Amyloid deposition, HC = Healthy Control, DLB = Dementia with Lewy Bodies, FTD = Frontal Temporal Dementia.

The distribution of the scores given to each subjects’ images is shown in Fig. 3. In general, all images showed a statistically significant difference in the SSM/PCA scores between groups (ANOVA, *p* < 0.05). After correcting for multiple comparisons, the *R*_1_ scores (Fig. 3, top left) were significantly different between AD and MCI+ (difference between groups: 1.36, CI of [0.33; 2.4], *p* < 0.01), MCI− (1.32, [0.16; 2.48], *p* = 0.01), and HC (1.58, [0.61; 2.54], *p* < 0.01) groups. The FDG-SUVR scores (Fig. 3, top right) from the AD group were significantly different from the MCI+ (1.28, [0.15; 2.41], *p* = 0.2), MCI- (1.63, [0.36; 2.89], *p* < 0.01), and HC (2.27, [1.23; 3.32], *p* < 0.01) groups, but not for the DLB and FTD; the HC group was significantly different from the DLB (−2.28, [0.69; 3.86], *p* < 0.01) and FTD (−1.98, [0.29; 3.68], *p* = 0.01) scores. The AD and MCI+ groups presented no significant difference between each other in the *BP*_ND_ (Fig. 3, bottom left) and PIB SUVR (Fig. 3, bottom right) scores, however, they were distinct from all the remaining groups. For the *BP*_ND_, the difference for the AD group mean with the MCI− was of 6.87, and the CI [4.47; 9.26]; with the HC was 7.92, [5.94; 9.91]; with DLB, of 7.70, [4.79; 10.6]; and with FTD, of 7.64, [4.56; 10.81]. Meanwhile, the MCI+ group had a difference of 7.64 and CI of [5.01; 10.28] with the mean score of the MCI− group; of 8.70 and [6.43; 10.97] with the HC; of 8.47 and [5.37; 11.58] with the DLB; and of 8.46 and [5.15; 11.78] with the FTD. These comparisons resulted in a *p* < 0.01. For the amyloid scores, the AD group was significantly different from the MCI− (6.52, [4.23; 8.80], *p* < 0.01), HC (7.40, [5.51; 9.29], *p* < 0.01), DLB (7.26, [4.49; 10.03], *p* < 0.01), and FTD (8.2, [4.49; 10.46], *p* < 0.01). The MCI+ group was also significantly different from the MCI− (7.24, [4.72; 9.75], *p* < 0.01), HC (8.12, [5.96; 10.29], *p* < 0.01), DLB (7.98, [5.02; 10.95], *p* < 0.01), and FTD (8.20, [5.04; 11.36], *p* < 0.01). Means, standard deviations, and range of the scores for all groups in all methods can be found in Table 2.

**Table 2:** Mean, SD, and range of Z-scores per diagnosis for each type of image used in the analysis.

Image	Diagnosis	Mean	SD	Range
PIB-*R*_1_	AD	1.58	1.21	[−1.69; 3.95]
	MCI+	0.21	1.05	[−1.21; 1.82]
	MCI−	0.25	1.13	[−1.08; 2.13]
	HC	0.00	1.00	[−1.52; 2.39]
	DLB	1.02	0.61	[0.29; 2.00]
	FTD	1.07	0.40	[0.59; 1.69]

FDG-SUVR	AD	2.27	1.21	[−0.10;5.79]
	MCI+	0.99	0.99	[−0.78; 3.17]
	MCI−	0.65	1.74	[−2.47; 3.32]
	HC	0.00	1.00	[−1.35; 2.65]
	DLB	2.28	0.68	[1.41; 3.21]
	FTD	1.98	0.40	[1.49; 2.57]

PIB-*BP*_ND_	AD	7.92	2.88	[−0.08; 12.30]
	MCI+	8.70	2.80	[4.09; 14.01]
	MCI−	1.06	1.44	[−0.40; 4.43]
	HC	0.00	1.00	[−1.49; 2.41]
	DLB	0.23	0.90	[−0.63; 1.56]
	FTD	0.24	0.90	[−0.50; 1.75]

PIB-SUVR	AD	7.40	2.88	[−0.08; 12.30]
	MCI+	8.12	2.80	[4.09; 14.01]
	MCI−	0.89	1.44	[−0.40; 4.43]
	HC	0.00	1.00	[−1.49; 2.41]
	DLB	0.14	0.90	[−0.63; 1.56]
	FTD	−0.08	0.90	[**−**0.50; 1.75]

## 4 Discussion

The primary aim of this study was to explore the feasibility of using quantitative PET pharmacokinetic parametric images as input for SSM/PCA analysis, using as an example the images derived from dynamic PIB PET scans. A secondary aim was to explore to what extent results obtained with these images correlated with the ones obtained using semi-quantitative SUVR images, which are more common in the clinical setting. The SSM/PCA technique allows the generation of a characteristic DP that can be used to test new subjects and derive a score, which reflects their similarity with the DP and could potentially be used for clinical assessment. So far, this approach has been used mainly for FDG PET scans (Meles et al., 2017, Spetsieris et al., 2009, Spetsieris and Eidelberg, 2011, Teune et al., 2014b, Teune et al., 2010). However, quantitative pharmacokinetic parametric images of other radiotracers might be used as an input for this analysis as well, with the advantage of providing additional and more accurate information than semi-quantitative images. Multiple parametric images derived from a single dynamic PET scan provide more information (flow and uptake) than a single static scan (Lammertsma, 2017). Hence a single dynamic scan might reduce the need of a second FDG PET study.

Both *R*_1_ and FDG-SUVR DPs presented a general cortical decrease in flow and metabolism, respectively, in AD patients when compared to HC subjects. Since flow and metabolism are related (Jueptner and Weiller, 1995), similarity between the two patterns was expected. Yet some differences between the two were observed in the brainstem, thalamus, cerebellum, and occipital lobe. Hyperperfusion is known to occur in these first three regions (Gur et al., 2009), and they were, therefore, more positively expressed in the *R*_1_ DP than in the FDG-SUVR DP. This effect has been observed before when comparing regional metabolic and flow values (Peretti et al., 2019c); these regions presented higher average flow values than metabolic uptake. In contrast, the occipital lobe showed the opposite effect: it was more pronounced in the metabolism than in the flow pattern. AD is a disease known not to affect the occipital lobe in most of patients and, therefore, it might be assumed that the metabolism of this brain region is not strongly altered in this disease.

Both *BP*_ND_ and PIB-SUVR DPs showed a general increase in tracer binding in cortical grey matter. The patterns reflect what is already known about the differences between AD and HC: that the first shows a large amyloid deposition across brain cortex in comparison with the latter. It is interesting to notice that some regions, such as the parietal, temporal and frontal lobes, seem to show more Aβ deposition than others (Braak and Braak, 1991). These regions have been shown to be the first ones to be affected by Aβ deposits and, therefore, present higher deposition in later stages of the disease (Grothe et al., 2017). Furthermore, the occipital lobe seems to be much less affected by amyloid plaques than the rest of the cortical matter, as was also seen in the flow and metabolism patterns (Peretti et al., 2019c). The shape of the joint histogram comparing the *BP*_ND_ and PIB-SUVR DPs shows the similarity of the two patterns.

The distribution of HC score in Fig. 3 suggests that the reduction in flow of healthy elderly subjects is smaller than the reduction in metabolism, making the distinction between HC and AD subjects less sensitive. This is consistent with the smaller spread of scores for *R*_1_ when compared to FDG-SUVR. Moreover, the same figure shows that the DLB and FTD subjects obtain similar scores to AD patients both in metabolism and flow (even if they are known not to have the same pattern (Diehl-Schmid et al., 2007)), but not in *BP*_ND_ and PIB-SUVR. This suggests that the combined information provided through *BP*_ND_ and *R*_1_ from a single dynamic scan will be sufficient to distinguish AD patients not only from HC subjects, but also from subjects with other neurodegenerative disorders. Fig. 3 shows that most MCI+ subjects have a score above the AD classification threshold for *R*_1_, FDG-SUVR, *BP*_ND_, and PIB-SUVR. Since MCI+ is part of the Alzheimer spectrum and is a prodromal stage of Alzheimer (in fact, these patients are classified as MCI due to AD), it could be speculated that these subjects are more likely to convert than other patients from the same group. However, to confirm such an affirmation, a follow up study of these patients is needed.

The use of a single tracer study to assess both amyloid deposition (through *BP*_ND_ or SUVR) and rCBF is a strong advantage of using pharmacokinetic modelling of dynamic PIB PET scans. For patients, the main benefit is that both imaging biomarkers can be obtained during one rather than two imaging procedures, thus minimizing the number of visits as well as radiation exposure. For initial differential diagnosis, the use of flow with *BP*_ND_ from a single dynamic study might suffice, allowing for a reduction in patient radiation exposure and discomfort, study cost, and visits. Despite the high AUC for the distinction between AD and HC subjects using *R*_1_, rCBF might be less sensitive to small changes than those seen with FDG (Peretti et al., 2019c). Therefore, if there is still doubt about a subject’s diagnosis based on *R*_1_ and *BP*_ND_, a PET scan using FDG may be considered for confirmation. Although the sensitivity and specificity found in this study are good when comparing the SSM/PCA Z-scores with the visual assessment by the clinicians, a more precise estimation of these values could be found with a larger independent dataset. Finally, SSM/PCA can be used to test a single subject against a characteristic pattern which allows its use in the clinic and can reduce the variability of visual reads, which are dependent on reader’s experience (Herholz et al., 2002).

In this study, all analyses were performed using PIB as a radiotracer. Nonetheless, it can be assumed that similar results may be found for other amyloid tracers, such as [^18^F]Florbetapir, [^18^F]Florbetaben, and [^18^F]Flutemetamol, since the target for tracer binding is the same (Morris et al., 2016). Further research to validate parametric maps derived from dynamic ^18^F-labelled amyloid tracers are still required. Moreover, this study was done with a modest sample of subjects, which might have limited the accuracy of the specificity and sensitivity of the DPs. Still, very promising results were found, justifying further exploration of SSM/PCA for other amyloid tracers and for other diseases. Furthermore, it might be interesting to use a longitudinal dataset to evaluate its ability to measure disease progression and to predict conversion to AD for patients at high risk (MCI+). Additional research is needed to evaluate the use of *R*_1_ parametric images as these might not be sensitive enough to detect small changes during a follow-up of a patient (Peretti et al., 2019b, Peretti et al., 2019c). Moreover, all images were only tested against an AD DP. It might be interesting to generate a DP for each of the diseases, so that the image of a subject can be compared to all of them. This could potentially help with the differential diagnosis of a patient in a clinical setting. However, the dataset of this study did not have enough subjects to generate a DP per disease and, therefore, this should be explored by another study.

The SSM/PCA approach has been in use in research settings since 2009 (Spetsieris et al., 2009), yet its optimization was performed for a group of Parkinson’s disease subjects. It might be interesting to further adapt the SSM/PCA to each specific research setting. Although this technique already provides a good separation between subjects, better results might be achieved by using different settings. The predefined steps used by the SSM/PCA for data reduction and combination of components could be exchanged by ICA (Pagani et al., 2017, Toussaint et al., 2012) and a decision tree (Mudali et al., 2015). Finally, the SSM/PCA approach can be used in a clinical setting once biomarker specific disease profiles are generated, as shown in this study. Newly imaged subjects can then be easily and quickly assessed by estimating the expression of each DP in their images and thereby to assisting in making a differential diagnosis. With the modifications investigated in this study, different types of images can be used, increasing the range of applications of the SSM/PCA in clinical settings.

In conclusion, *R*_1_ and *BP*_ND_ parametric images can be used as input for an SSM/PCA analysis using pharmacokinetic modelling of a single dynamic PIB PET scans with the classification of AD and HC as a case control task provided small changes are made in the steps used for the analysis. Moreover, using the example of PIB-PET parametric images, the DPs generated by these images provided a good classification and offered complementary information for differentiation of other neurodegenerative disorders than could not be achieved with a single FDG PET scan, reinforcing results found in previous studies.

## Author contribution statement

D.E.P., D.V.G., and R.B. were responsible for study design. F.E.R., P.P.D.D., and R.B. coordinated the study. D.E.P. and D.V.G. were responsible for image processing and analysis. R.J.R., D.V.G., and D.E.P. were responsible for the development and maintenance of the software used in this study. D.E.P., D.V.G., and R.B. were responsible for the initial draft of the manuscript. All authors critically revised the final version of the manuscript.

## Disclosures

Ronald Boellaard has received funding from the European Union’s Horizon 2020 research and innovation programme under the Marie Skłodowska-Curie grant agreement No. 764458, which is not related to this work.

## CRediT authorship contribution statement

**Débora E. Peretti:** Conceptualization, Data curation, Formal analysis, Investigation, Methodology, Software, Validation, Writing - original draft, Writing - review & editing.

## Declaration of Competing Interest

The authors declare that they have no known competing financial interests or personal relationships that could have appeared to influence the work reported in this paper.
